# Dual-target tDCS and dual-task training modulate neuroinflammation and neuroplasticity: transcriptomic and behavioral evidence in stroke rehabilitation

**DOI:** 10.3389/fresc.2025.1589588

**Published:** 2025-10-17

**Authors:** Yutong Fu, Qianxi Yan, Anjuan Wang, Hongmei Zhang, Wenli Wang, Liqing Yao, Devinder Kaur Ajit Singh

**Affiliations:** 1 Department of Rehabilitation Medicine, The Second Affiliated Hospital of Kunming Medical University, Kunming, China; 2 Center for Healthy Ageing and Wellness (H-CARE), Faculty of Health Sciences, Universiti Kebangsaan Malaysia, Kuala Lumpur, Malaysia

**Keywords:** tDCS, dual-task training, neurorehabilitation, transcriptomics, inflammation, neuroplasticity

## Abstract

**Background:**

Transcranial direct current stimulation (tDCS) combined with dual-task training (DTT) has shown potential in promoting neurorehabilitation. However, the transcriptomic mechanisms underlying the synergistic effects of dual-target tDCS remain unexplored. This study aims to evaluate the effects of tDCS + DTT on cognitive and motor functions and preliminarily explore its molecular basis through transcriptomic analysis.

**Methods:**

Fifty two chronic stroke patients were randomized to receive dual-target tDCS (anodal electrodes over affected primary motor cortex M1 and left dorsolateral prefrontal cortex DLPFC) combined with DTT (*n* = 26) or sham stimulation with DTT (*n* = 26). Behavioral assessments, including the Visual Cognitive Assessment Test (VCAT), Hamilton Depression Scale (HAMD), Fugl-Meyer Lower Limb Assessment (FMA-L), Timed Up and Go Test (TUG), and Modified Barthel Index (MBI), were conducted before and after the intervention. Peripheral blood transcriptomic analysis was performed on a subset of patients from the tDCS + DTT group to identify differentially expressed genes (DEGs) and enriched pathways.

**Results:**

Significant interactions were observed for VCAT (*p* < 0.001), MBI (*p* = 0.033), HAMD (*p* < 0.001), FM-L (*p* < 0.001), TUG-CMDT time (*p* < 0.001), and TUG-CMDT accuracy rate (*p* < 0.001). Transcriptomic analysis revealed 1,319 DEGs post-treatment, predominantly downregulating inflammation/apoptosis-related genes (1,155) and upregulating neuroplasticity-associated genes (164). KEGG pathway analysis highlighted suppressed NF-κB signaling and apoptosis pathways, alongside enhanced synaptic plasticity mechanisms. Key regulatory genes, such as PPP1R15A, BCL3, GADD45B, and NFKBIA, were identified as potential mediators of tDCS-induced neuroprotection.

**Conclusion:**

Dual-target tDCS combined with DTT promotes functional recovery in stroke patients through transcriptomic reprogramming of inflammatory and neuroplastic pathways, offering a novel strategy for multi-modal neurorehabilitation.

## Introduction

Stroke is the second leading cause of death globally, accounting for 11.6% of global mortality (1), and the third leading cause of disability, with over 80 million stroke survivors worldwide. These survivors often experience a range of disabilities, including motor dysfunction (2), cognitive decline (3), language impairments (4), mood disorders, and diminished activities of daily living (5).

Stroke-related disability is especially prevalent in low-income and developing regions (6). With the aging population, the incidence of stroke is projected to steadily rise, and by 2030, the annual medical costs associated with stroke are expected to exceed $183 billion (7). As such, improving rehabilitation outcomes, shortening rehabilitation timelines, and exploring more effective rehabilitation approaches are pressing issues in both medical research and clinical practice.

Transcranial direct current stimulation (tDCS) combined with DTT has gained significant attention in recent years for its synergistic effects in promoting brain function recovery in different nervous disease rehabilitation, like Parkinson (8), stroke (9) and Alzheimer's (10). However, the precise underlying regulatory mechanisms of this combined approach remain unclear.

This study aims to systematically explore the mechanisms of action of tDCS combined with DTT in stroke rehabilitation, employing multimodal assessments including behavioral analysis and transcriptomic sequencing. The goal is to provide theoretical support for personalized rehabilitation strategies and advance the understanding of stroke recovery mechanisms.

tDCS has emerged as a promising non-invasive brain stimulation technique that modulates cortical excitability, enhancing neuroplasticity and promoting functional recovery in individuals with neurological conditions, including stroke (11). By applying a low electrical current to the scalp, tDCS promotes cortical reorganization and induces transcriptomic changes associated with synaptic plasticity and neuroprotection (12). While several studies have demonstrated the efficacy of tDCS in improving motor and cognitive outcomes in stroke patients, the underlying transcriptomic changes induced by tDCS remain insufficiently understood (13). Specifically, there is a need to explore how tDCS influences gene expression related to neuroplasticity, inflammation, and tissue regeneration in the context of stroke rehabilitation.

In parallel, DTT has gained attention as an effective rehabilitation approach, which simultaneously challenges both cognitive and motor functions by engaging multiple brain networks (14). DTT has been shown to improve functional outcomes by promoting cognitive-motor integration, brain reorganization, and compensatory strategies (15). Emerging evidence highlights that dual-target tDCS over primary motor cortex (M1) and dorsolateral prefrontal cortex (DLPFC) cortices synergistically enhances cognitive-motor integration by modulating large-scale brain networks (31). This approach aligns with the central-peripheral-central closed-loop theory, which posits that combined neuromodulation and task-specific training optimize neural circuit reorganization. According to the Central-peripheral-central (CPC) closed loop (16), when combined with DTT and tDCS, this strategy has the potential to enhance the efficacy of traditional rehabilitation protocols. However, while there is a growing body of evidence supporting the benefits of DTT, little is known about the combined effects of tDCS and DTT on molecular processes and their correlation with functional recovery.

This study aims to investigate the synergistic effects of tDCS and DTT on functional recovery in stroke patients, with a specific focus on the transcriptomic changes associated with these interventions. We hypothesize that the combination of tDCS and DTT will lead to significant alterations in gene expression related to neuroplasticity, neuronal repair, and neuroprotection, which will correlate with improvements in both motor function and cognitive performance.

By integrating tDCS and DTT, this study seeks to bridge the gap between neurostimulation and cognitive-motor rehabilitation, offering a comprehensive approach to stroke recovery. The findings from this preliminary investigation will provide critical insights into the molecular mechanisms underpinning neuroplasticity and functional recovery following combined tDCS and DTT interventions, paving the way for future clinical applications and studies in stroke rehabilitation.

## Materials and methods

### Participants

This study included 52 adult stroke patients who were recruited from the Rehabilitation Department of the Second Affiliated Hospital of Kunming Medical University Hospital. There were 17 female, 35 male in two groups in total. Demographic data of participants as shown in Table 1. There were no significant difference between sociodemographic, cognitive, physical ADL, and mood parameters between the tDCS + DTT group and Sham + DTT group (*p* > 0.05; Table 1).

**Table 1:** Demographic data of participants.

Sociodemographic and clinical data	A-tDCS + DTT *n* = 26	Sham + DTT *n* = 26	*P*
Sociodemographic			
Age (years)	53.30 ± 1.97	53.84 ± 2.31	0.86
Onset (months)	8.40 ± 1.47	8.44 ± 1.74	0.92
Gender (male/female)	17/9	18/8	0.76
Affected side (left/right)	11/15	13/13	0.57
Type (Hemorrhage/ischemic)	11/15	13/13	0.57
Cognitive function			
Moca (score)	17.34 ± 6.02	18.92 ± 5.30	0.32
VCAT (score)	19.88 ± 5.84	22.23 ± 5.39	0.13
Physical function			
TUG-CMDT (walking and 100-3) time (seconds)	50.29 ± 22.07	48.27 ± 21.43	0.73
TUG-CMDT (walking and 100-3) time (accuracy rate)	34.24 ± 23.30	31.89 ± 20.65	0.70
FM-L (score)	20.11 ± 6.62	22.34 ± 8.25	0.28
ADL			
MBI (score)	79.23 ± 14.40	82.69 ± 13.35	0.37
Psychology			
HAMD (score)	7.34 ± 3.71	8.26 ± 4.18	0.40

Inclusion criteria were: (1) diagnosis of ischemic or hemorrhagic stroke confirmed by neuroimaging, middle cerebral artery region, (2) first-ever stroke, (3) stroke onset within 6–12 months from the start of the intervention (4) MoCA score between 7 and 26, (5)age between 18 and 80 (6) ability to follow instructions and provide informed consent.

Exclusion criteria included: (1) history of any prior stroke (clinical or radiologically confirmed),(2) neurological disorder other than stroke, (3) severe speech impairment, (4) severe pain during exercise or rest, (5) mental disease, (6) cardiovascular diseases that affect rehabilitation, such as heart failure, (7) severe visual or hearing impairment, (8) any contraindications to tDCS, (9) severe musculoskeletal disease.

### Study design

This was a randomized controlled trial designed to investigate the effects of combined tDCS and Dual Task Training (DTT) on functional recovery in stroke patients. Participants were randomly assigned to either the tDCS + DTT intervention group or a sham control group. The intervention lasted for 2 weeks, with sessions conducted six sessions a week for two weeks.

### Intervention protocol

#### Dual-target tDCS protocol

tDCS was administered using the IS200 stimulator (Sichuan Intelligent Electronics, China) with two anodal electrodes (5 × 7 cm gelatin sponge) positioned over the affected primary motor cortex (M1) and left dorsolateral prefrontal cortex (DLPFC), localized via the 10–20 EEG system (M1: C3/C4 contralateral to the affected hemisphere; DLPFC: F3). The cathodal electrode (5 × 7 cm) was placed over the contralateral supraorbital area (FP2) to minimize off-target effects.

Stimulation parameters included:

Current intensity: 2 mA
Duration: 30 min/session
Frequency: 6 sessions/week for 2 weeks

### Sham tDCS protocol

The sham group received identical electrode placement with 30-s ramp-up/down phases and no sustained current, ensuring participant blinding.

### Participant randomization and group allocation

#### Sample size and randomization

Fifty two stroke patients (17 female, 35 male; age: 53.30 ± 1.97 vs. 53.84 ± 2.31 years in active vs. sham groups) were randomly allocated using block randomization (1:1 ratio) to either:

Active tDCS + DTT group (*n* = 26): Received dual-target tDCS (M1 + DLPFC) combined with dual-task training.
Sham tDCS + DTT group (*n* = 26): Received sham stimulation with identical DTT.

#### Blinding and baseline comparability

Participants and outcome assessors were blinded to group allocation. Baseline characteristics (age, MoCA scores, stroke onset time) showed no significant differences between groups (all *p* > 0.05; Table 1), ensuring initial comparability.

The rehabilitation therapists who delivered the DTT intervention were also blinded. The technicians who set up the tDCS device could not be blinded because they had to adjust current intensity, but they had no role in outcome assessment or clinical care.

Assessment time-points were identical for all participants: baseline evaluations were completed within 24 h before the first session and follow-up evaluations within 24 h after the final (12th) session.

### Dual-task training (DTT) protocol

#### Task design and progression

DTT sessions involved simultaneous motor-cognitive challenges, such as:

Walking while performing serial subtraction (100-3, 97-3, etc.)
Balancing on uneven surfaces while responding to auditory cues

Tasks were adapted weekly based on performance (e.g., increasing cognitive load or motor complexity) to maintain a 70%–80% success rate. Each session lasted 40 min, delivered 6 days/week for 2 weeks.

#### Safety monitoring

All participants were closely monitored for any adverse effects related to tDCS during the intervention. Adverse events, including skin irritation, dizziness, or discomfort, were recorded and assessed in accordance with established safety protocols. Any serious adverse events were reported to the ethics committee immediately.

#### Regular rehabilitation training

Participants receive single task based on their evaluation outcomes. Physical, occupational or speech therapy conducted by professional therapist once a day, in total of 12 sessions for two weeks.

### Outcome measures

#### Behavioral assessment

Functional recovery was assessed using a battery of standardized tests, including Fugl-Meyer Assessment lower limb (FM-L), Timed Up and Go test (TUG).

#### Cognition assessment scale

A Visual cognitive assessment test (VCAT).

#### Activities of daily index

Modified Barthel Index (MBI).

#### Psychological assessment

Hamilton Depression Scale (HAMD).

These assessments were conducted at baseline and post- intervention.

#### Transcriptomic analysis

Blood samples were collected at baseline and after the intervention period for transcriptomic analysis. RNA extraction was using Trizol reagent (Invitrogen, CA, USA), and the quality and quantity of RNA were assessed using NanoDrop. RNA sequencing was conducted using Illumina to examine changes in gene expression associated with neuroplasticity, inflammation, and neuroprotection. The data were analyzed using DESeq2 to identify differentially expressed genes between pre- and post-intervention samples.

### Statistical analysis

Data were analyzed using SPSS23.0 for behavioral data, descriptive statistics were generated for all variables. Variable distributions that conformed to the normal distribution were expressed as mean ± standard deviation, while counting data are expressed in terms of frequency. The differences in baseline characteristics between groups were analyzed by correlation *t*-test or chi-square test. A repeated measures ANOVA was conducted with time (pre-test, post-test) as the within-subject factor and group (intervention group, control group) as the between-subject factor. For transcriptomic data, DESeq2 were used to identify genes with significant changes in expression between time points. A significance level of *p* < 0.05 was used for all statistical tests.

## Results

Baseline characteristics between the tDCS + DTT group (*n* = 26) and sham + DTT group (*n* = 26) demonstrated no significant differences (all *p* > 0.05), ensuring initial group comparability in Table 1. After 12 sessions of treatment, a repeated ANOVA was conducted to compare the pre-, post-and within group cognitive, physical ADL and mood outcomes.

Table 2 was showen about main effect of time, group, and time–group interaction of the interventions on the physical, cognitive, ADL and mood functions. VCAT revealed significant effects of time (*p* < 0.001) and group (*p* = 0.911), with a significant interaction effect (*p* < 0.001). MBI scores indicated significant effects of time (*p* < 0.001), group (*p* = 0.27), and interaction (*p* = 0. 33). HAMD scores revealed significant effects of time *(p* < 0.001), group (*p* < 0.000), and interaction (*p* < 0.001). FM-L scores showed significant effects of time (*p* < 0.001), group *(p* = 0.86), and interaction (*p* < 0.001). TUG-CMDT time revealed significant effects of time (*p* < 0.001), group (*p* = 0.20), and interaction (*p* < 0.001). TUG-CMDT accuracy rate results showed significant effects of time (*p* < 0.001), group (*p* = 0.30), and interaction (*p* < 0.001).

**Table 2:** Main effect of time, group, and time–group interaction of the interventions on the physical, cognitive, ADL and mood functions.

Parameters	Study group		Analysis of covariance (*p*-value)		
	Experimental (Mean ± SD)	Control (Mean ± SD)	Time (*η*p^2^)	Group (*η*p^2^)	Interaction (*η*p^2^)
Cognitive measure					
VCAT (score)					
Week 0	19.88 ± 5.84	22.23 ± 5.39	<0.001 (0.73)	0.91 (0.00)	<0.001 (0.41)
Week 2	27.38 ± 4.04	24.73 ± 4.92			
MBI (score)					
Week 0	79.23 ± 14.40	82.69 ± 13.35	<0.001 (0.40)	0.27 (0.24)	0.33 (0.19)
Week 2	82.11 ± 13.42	86.73 ± 12.32			
HAMD (score)					
Week 0	7.34 ± 3.71	8.26 ± 4.18	<0.001 (0.59)	<0.001 (0.13)	**<0.001** (0.30)
Week 2	2.88 ± 1.88	7.00 ± 3.55			
Physical function					
FM-L (score)					
Week 0	20.11 ± 6.62	22.34 ± 8.25	<0.001 (0.69)	0.86 (0.00)	**<0.001** (0.45)
Week 2	26.84 ± 5.06	23.96 ± 7.82			
TUG-CMDT (walking and 100-3) (seconds)					
Week 0	50.29 ± 22.07	48.27 ± 21.43	<0.001 (0.44)	0.20 (0.03)	<0.001 (0.22)
Week 2	28.49 ± 9.66	42.66 ± 18.97			
TUG-CMDT (walking and 100-3) (accuracy)					
Week 0	34.24 ± 23.30	31.89 ± 20.65	<0.001 (0.59)	0.30 (0.02)	**<0.001** (0.27)
Week 2	46.78 ± 26.97	35.98 ± 21.48			

Bold values in the table indicate statistical significance at the *p* < 0.05 level. Specifically, these values highlight the results where the differences between the experimental and control groups, across different time points, are considered to be statistically significant according to the analysis of covariance (ANCOVA) with the corresponding *p*-values.

### Transcriptomic changes

**Figure 1:**
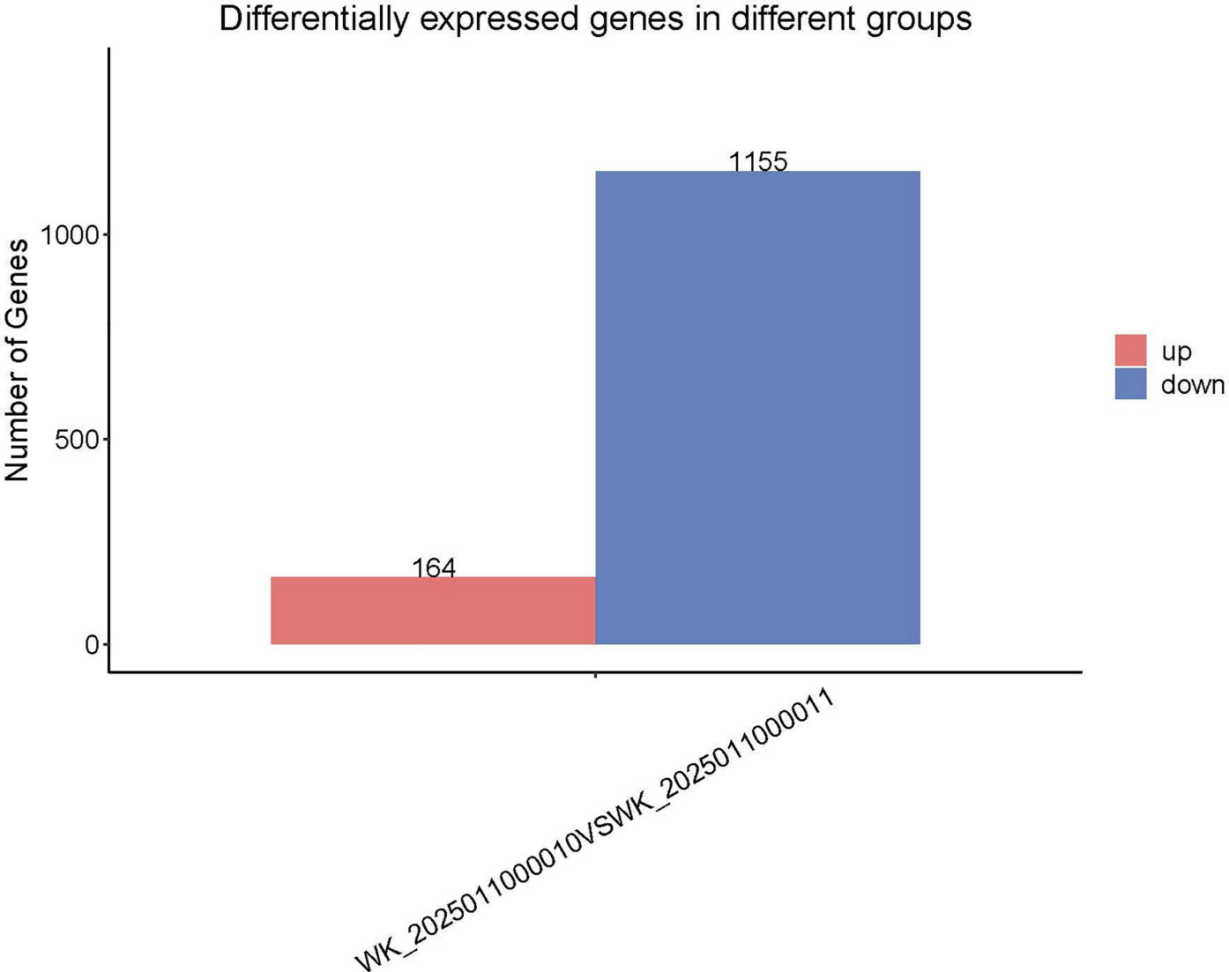
Volcano plot illustrating differentially expressed genes before and after tDCS + DTT treatment. Red dots indicate significantly upregulated genes, while blue dots represent significantly downregulated genes (*p* < 0.05, |log2FC| > 1), FDR < 0.05.

Transcriptomic Profiling Reveals Inflammation Suppression and Neuroplasticity Enhancement RNA sequencing of peripheral blood from tDCS + DTT-treated patients identified 1,319 differentially expressed genes (DEGs) (FDR < 0.05), with two distinct functional clusters (Figure 1):

1. Downregulated Inflammation/Apoptosis Network: 1,155 genes showed decreased expression, primarily enriched in pro-inflammatory pathways (e.g., NF-κB signaling, cytokine-cytokine receptor interaction) and apoptotic regulation (e.g., BCL3, GADD45B).
2. Upregulated Neuroplasticity Network: 164 genes exhibited increased expression, linked to synaptic plasticity (e.g., BDNF, SYN1) and neuroprotection (e.g., NFKBIA, PPP1R15A).

**Figure 2:**
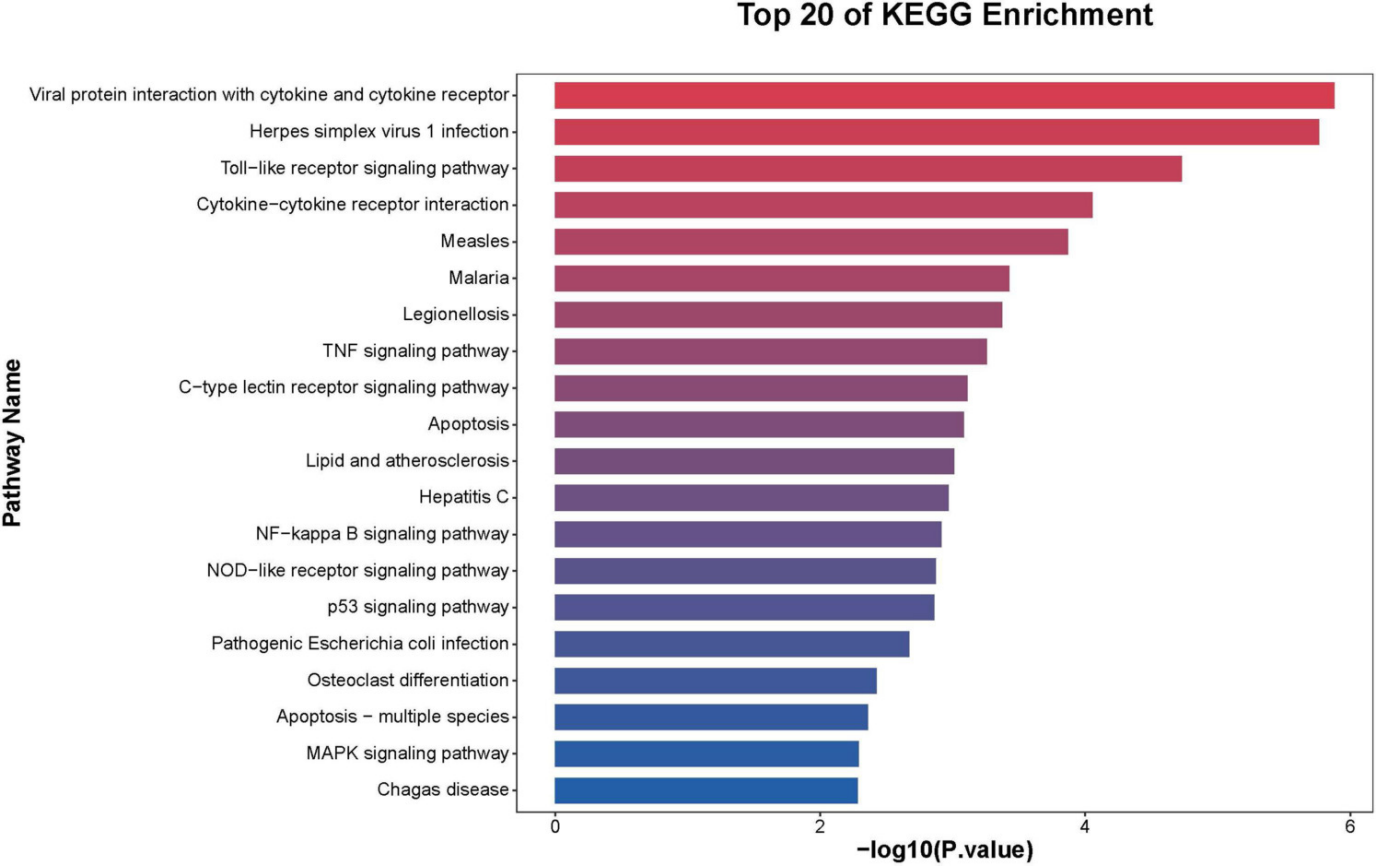
KEGG pathway enrichment analysis of differentially expressed genes. The pathways are ranked based on their enrichment scores, with a strong association observed in immune and inflammatory regulatory mechanisms.

**Figure 3:**
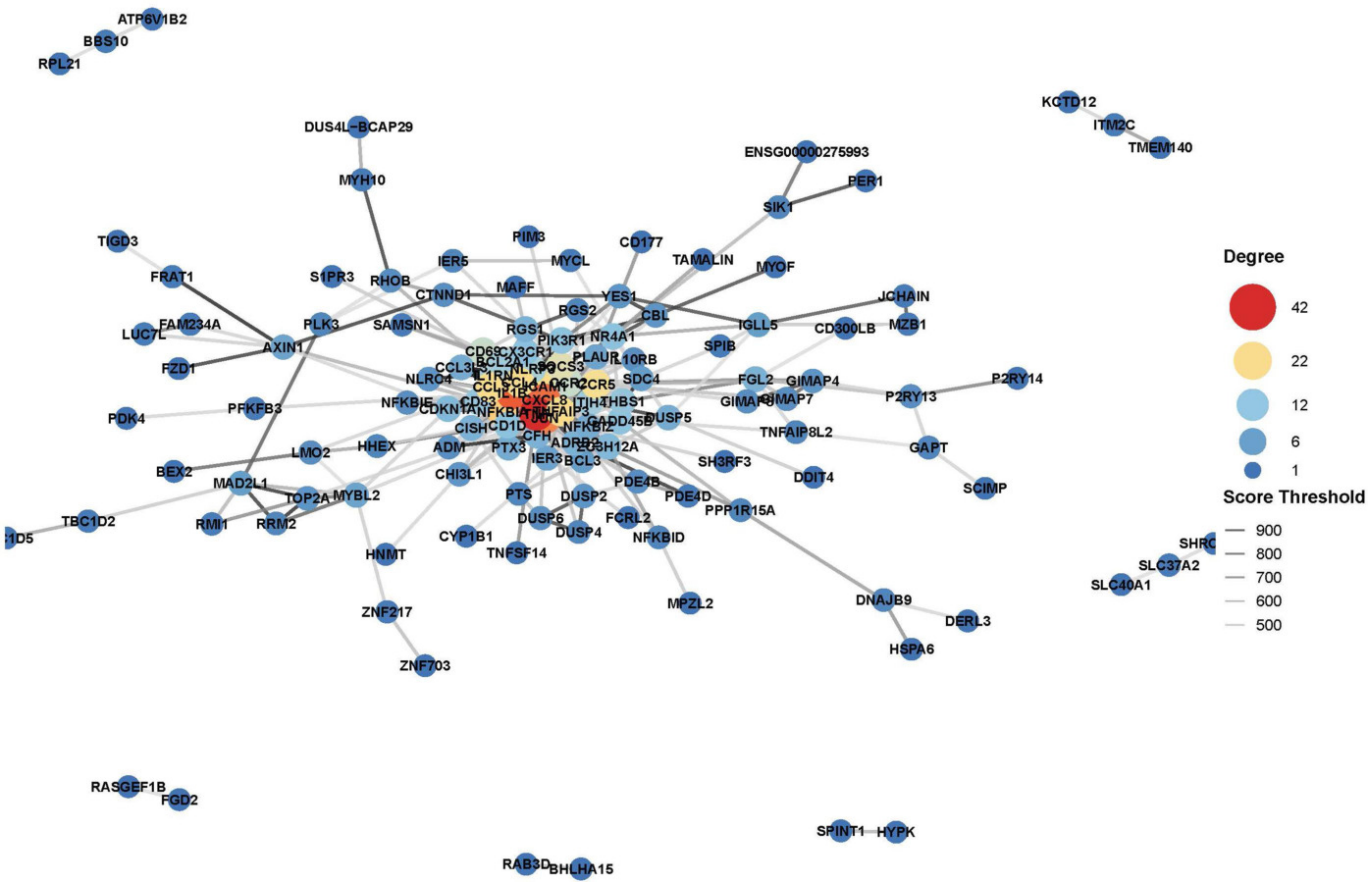
Gene-disease association analysis network.

**Figure 4:**
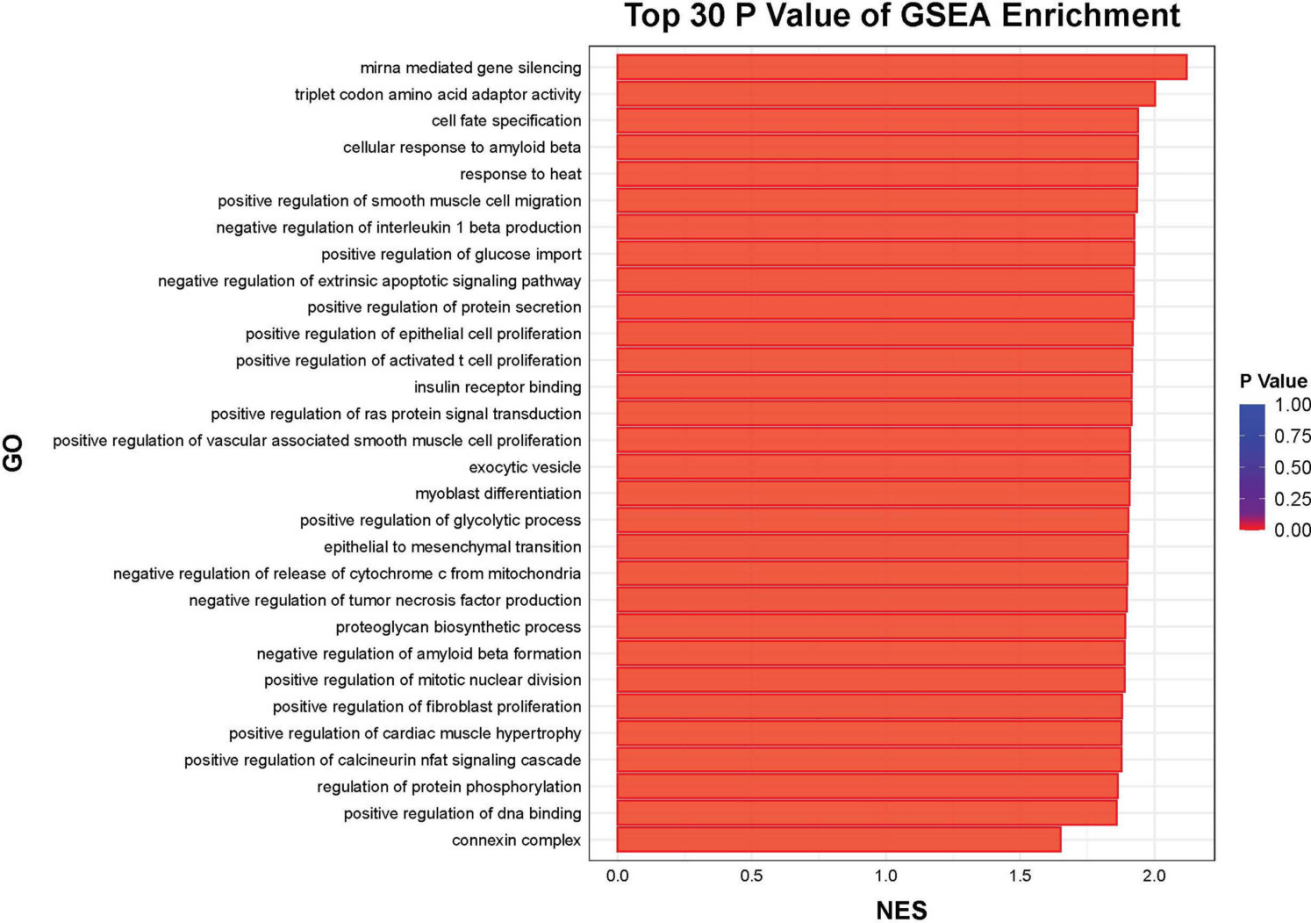
Top 30 GSEA enrichment.

Further functional enrichment analysis revealed that downregulated genes dominated the transcriptomic changes, leading to the suppression of multiple biological processes, including inflammatory responses, cell proliferation, and differentiation. Among the top 20 enriched pathways, significant enrichment was observed in Toll-like receptor signaling, cytokine–cytokine receptor interactions, and apoptosis-related pathways in Figure 2. Additionally, gene-disease association analysis identified 231 genes linked to motor neuron diseases and 8,725 genes associated with vascular dementia, highlighting the potential of tDCS + DTT in treating not only stroke-related impairments but also neurodegenerative conditions affecting both motor and cognitive functions. Key inflammation-related genes, including PPP1R15A, BCL3, GADD45B, NFKBIA, were found to form a tightly connected regulatory network, suggesting their critical roles in mediating the treatment effects in Figure 3. Furthermore, pathway analysis revealed links to β-amyloid metabolism, suggesting that tDCS + DTT might influence β-amyloid production and clearance, potentially contributing to cognitive improvements in neurodegenerative conditions in Figure 4.

## Discussion

This study provides compelling evidence that dual-target tDCS combined with DTT significantly enhances cognitive, motor and mood functions in chronic stroke patients. Our behavioral assessments demonstrated superior improvements in the tDCS + DTT group across multiple domains, including cognitive ability (VCAT), mood (HAMD), lower limb motor function (FMA-L), and functional mobility (TUG). Crucially, this is the first study to provide transcriptomic insights suggesting that these functional gains are accompanied by profound molecular changes in peripheral blood during tDCS combined with DTT. Specifically, our exploratory analysis identified 1,319 differentially expressed genes post-treatment, predominantly showing downregulation of genes associated with inflammation, apoptosis, and neurodegeneration, alongside a clear upregulation of genes linked to synaptic plasticity and neuroprotection. These findings suggest that dual-target tDCS + DTT exerts its therapeutic effects through a multi-faceted approach involving active neuroplasticity modulation, neuroinflammation suppression, and synaptic remodeling, positioning it as a promising intervention for neurorehabilitation.

Our observed significant improvements in VCAT, HAMD, FMA-L, TUG, and MBI scores for the tDCS + DTT group are consistent with previous reports on the benefits of tDCS and DTT in stroke rehabilitation (17, 18). The comprehensive nature of these improvements, spanning cognitive, motor, and functional independence, underscores the broad therapeutic potential of this combined intervention. The superior efficacy of the dual-target tDCS + DTT approach compared to sham + DTT highlights the specific contribution of neuromodulation to enhancing recovery in this patient population.

A key novel finding of this study is the preliminary transcriptomic evidence of neuroinflammation suppression. Gene expression alterations suggest that tDCS + DTT may exert its effects by downregulating specific genes involved in neuronal excitability, synaptic function, and excitatory neurotransmission (19). A possible mechanism involves the modulation of neuronal synapses toward a more negative resting membrane potential, thereby facilitating excitatory responses through gene upregulation (20). These findings suggest that electrical stimulation may modulate inflammatory cytokines, suppress apoptosis, and regulate immune responses, potentially contributing to neuroprotection and functional recovery (21). The involvement of long-term depression and longevity regulation pathways further suggests a broader impact of this intervention on neuroplasticity and brain aging (22).

From our exploratory analysis, we observed a predominant downregulation of 1,155 genes associated with inflammation and apoptosis, with KEGG pathway analysis specifically highlighting suppressed NF-κB signaling and apoptosis pathways. Key regulatory genes such as PPP1R15A, BCL3, GADD45B, and NFKBIA were notably downregulated. This molecular signature aligns with prior evidence suggesting tDCS can reduce microglial activation and cytokine release in stroke models (23). By attenuating chronic inflammation, our intervention may help mitigate secondary neurodegeneration, a critical barrier to long-term stroke recovery (24). These initial findings provide a molecular basis for tDCS's neuroprotective effects and its potential in modulating disease progression.

Complementing the anti-inflammatory effects, our exploratory transcriptomic analysis also revealed the upregulation of 164 genes primarily linked to synaptic plasticity and neuroprotection. KEGG pathway analysis showed enhanced synaptic plasticity mechanisms. This upregulation of neuroplasticity-associated genes, including those involved in long-term potentiation (LTP) and synaptic reorganization, provides a molecular correlate for the behavioral improvements observed (13). These initial insights suggest that tDCS + DTT actively promotes neural circuit reorganization, thereby strengthening corticocortical connectivity and facilitating cognitive-motor integration, which is essential for functional recovery after stroke.

Simultaneously stimulating the affected M1 and the left DLPFC appears to leverage network-level modulation rather than isolated regional effects. The DLPFC plays a critical role in top-down cognitive control, attention, and executive functions. Its engagement by tDCS can enhance motor strategy adaptation and planning (25), which is particularly relevant for the cognitive demands of DTT. Concurrently, M1 stimulation boosts corticospinal excitability and facilitates skill consolidation (26). This multi-site stimulation approach may amplify functional connectivity between these regions (27), optimizing the neural network involved in complex cognitive-motor tasks. The heterogeneity in stroke patients' recovery (28) may further underscore the benefit of a broader, network-based approach like dual-target stimulation.

The combination of neuromodulation with active DTT is crucial. DTT itself challenges cognitive-motor integration, forcing the brain to adapt. The observed neuroplastic and anti-inflammatory changes at the transcriptomic level suggest that tDCS primes the brain for more effective learning and reorganization during the DTT sessions. This aligns with the central-peripheral-central closed-loop theory (16), which posits that integrating central (e.g., tDCS) and peripheral (e.g., task-specific training) interventions enhances brain plasticity through bidirectional feedback loops and sensorimotor interactions. This closed-loop strategy offers a comprehensive intervention that capitalizes on neuronal plasticity to restore neural repair and maximize functional recovery (29).

Prior studies have established that tDCS influences cortical excitability and promotes synaptic plasticity, contributing to cognitive and motor recovery (13, 30). However, our study significantly extends this understanding by integrating preliminary transcriptomic profiling, providing initial molecular-level insights into how dual-target tDCS + DTT drives functional recovery. Unlike prior research focusing solely on functional outcomes or limited mechanistic investigations, our exploratory findings illuminate the potential direct impact on gene expression related to both neuroinflammation and neuroplasticity. This initial transcriptomic evidence suggests that tDCS + DTT not only enhances functional recovery but also instigates crucial genetic and molecular changes underlying these improvements.

## Limitations

Despite its strengths in providing novel insights, this study has several limitations that warrant consideration, particularly regarding the transcriptomic data. Firstly, the transcriptomic analysis was performed on a small subset of the experimental group and utilized peripheral blood, which may not fully reflect gene expression changes directly within brain tissue. Crucially, no additional technical or biological replicates were included for these preliminary transcriptomic data. Therefore, these transcriptomic findings should be considered exploratory and hypothesis-generating rather than definitive evidence. This limits the statistical power and generalizability of the molecular insights presented. Secondly, our study focused on chronic stroke patients, so the generalizability of these findings to acute or subacute stroke phases requires further investigation. While the study was randomized and sham-controlled, the exact mechanisms by which these specific gene changes lead to functional improvements warrant more detailed investigation. Additionally, the long-term sustainability of the observed behavioral and transcriptomic changes needs to be assessed with extended follow-up periods.

## Clinical implications and future directions

Our findings strongly support the use of dual-target tDCS combined with DTT as a promising, multi-modal neurorehabilitation strategy for stroke patients. By demonstrating preliminary effects on both neuroinflammation and neuroplasticity at a molecular level, this intervention offers a novel approach to simultaneously address key pathological processes underlying post-stroke deficits. The identification of specific regulatory genes like PPP1R15A, BCL3, GADD45B, and NFKBIA as potential mediators opens new avenues for understanding tDCS-induced neuroprotection and could potentially serve as future biomarkers.

Given the exploratory nature of our initial transcriptomic data, future work is crucial to validate and expand upon these promising findings. We are currently undertaking studies in animal models using single-cell RNA-seq and multi-omics approaches, incorporating both technical and biological replicates to provide more definitive evidence of these molecular mechanisms. The results of these ongoing validation studies will be reported separately. Beyond validation, future research should also aim to optimize tDCS parameters, explore personalized approaches based on individual patient profiles, and conduct larger, multi-center human trials to further confirm these findings and facilitate their translation into widespread clinical practice.

## Data Availability

The datasets presented in this study can be found in online repositories. The names of the repository/repositories and accession number(s) can be found in the article/Supplementary Material.
