# Correction: Dual-target tDCS and dual-task training modulate neuroinflammation and neuroplasticity: transcriptomic and behavioral evidence in stroke rehabilitation

**DOI:** 10.3389/fresc.2025.1730113

**Published:** 2025-11-18

**Authors:** Yutong Fu, Qianxi Yan, Anjuan Wang, Hongmei Zhang, Wenli Wang, Liqing Yao, Devinder Kaur Ajit Singh

**Affiliations:** 1 Department of Rehabilitation Medicine, The Second Affiliated Hospital of Kunming Medical University, Kunming, China; 2 Center for Healthy Ageing and Wellness (H-CARE), Faculty of Health Sciences, Universiti Kebangsaan Malaysia, Kuala Lumpur, Malaysia

**Keywords:** tDCS, dual-task training, neurorehabilitation, transcriptomics, inflammation, neuroplasicity

**Author affiliation**

Affiliation 2 “Center for Healthy Ageing and Wellness (H-CARE), Faculty of Health Sciences, Universiti Kebangsaan Malaysia, Kuala Lumpur, Malaysia” was omitted for authors Yutong Fu and Devinder Kaur Ajit Singh. This affiliation has now been added for authors Yutong Fu and Devinder Kaur Ajit Singh.

**Error in author list**

Author Devinder Kaur Ajit Singh was omitted as an author in the published article. The correct author list reads:

Yutong Fu1,2, Qianxi Yan1, Anjuan Wang1, Hongmei Zhang1, Wenli Wang*1, Liqing Yao*1,Devinder Kaur Ajit Singh*2

1 Department of Rehabilitation Medicine, The Second Affiliated Hospital of Kunming Medical University, Kunming, China

2 Center for Healthy Ageing and Wellness (H-CARE), Faculty of Health Sciences, Universiti Kebangsaan Malaysia, Kuala Lumpur, Malaysia

The Author Contributions Statement has been corrected to read:

“YF: Conceptualization, Writing – original draft. QY: Data curation, Visualization, Writing – original draft. AW: Investigation, Writing – original draft. HZ: Software, Writing – original draft. WW: Supervision, Writing – review & editing. LY: Supervision, Writing – review & editing. DS: Conceptualization, Supervision, Writing – original draft, Writing – review & editing.”

**Corresponding author**

Author Devinder Kaur Ajit Singh is also a corresponding author. The correct corresponding authors are Wenli Wang, Liqing Yao, and Devinder Kaur Ajit Singh. The corrected Correspondence section appears below:

Wenli Wang, wangwenli0871@163.com

Liqing Yao, yaoliqing98731@163.com

Devinder Kaur Ajit Singh, devinder@ukm.edu.my

**Equal contribution statement**

Author Devinder Kaur Ajit Singh is an equal contributing author with Wenli Wang and Liqing Yao.

**Incorrect funding**

An incorrect funding number was provided for Yutong Fu. The correct number for Yunnan Provincial Rehabilitation Clinical Medical Center is zx2019-04-02, 2025KFZD007, and 2024YNLCYXZX0538.

The updated Funding appears below:

“The author(s) declare that financial support was received for the research and/or publication of this article. This research was funded by the Yunnan Provincial Rehabilitation Clinical Medical Center (zx2019-04-02, 2025KFZD007, and 2024YNLCYXZX0538 to YT), Neurorehabilitation Team of Kunming Medical University (2024XKTDTS18).”

**Ethics**

The ethics statement was erroneously given as: “The studies involving humans were approved by the Second Affiliated Hopital of Kunming Medical University. The studies were conducted in accordance with the local legislation and institutional requirements. Written informed consent for participation in this study was provided by the participants' legal guardians/next of kin.” The correct ethics statement is:

“The studies involving humans were approved by the Second Affiliated Hospital of Kunming Medical University and the Committee of Universiti Kebangsaan Malaysia. The studies were conducted in accordance with the local legislation and institutional requirements. Written informed consent for participation in this study was provided by the participants' legal guardians/next of kin.”

The original version of this article has been updated.

